# Development and characterization of a novel, small animal external beam irradiator using a clinical high dose rate brachytherapy source

**DOI:** 10.1002/mp.70540

**Published:** 2026-06-30

**Authors:** Daniel Cecchi, Sacha Freeman, Greg Warren, Brad Gill, Chris Johnstone, Devika B. Chithrani, Samantha A. M. Lloyd

**Affiliations:** ^1^ Department of Physics and Astronomy University of Victoria Victoria British Columbia Canada; ^2^ BC Cancer ‐ Victoria Victoria British Columbia Canada; ^3^ BC Cancer ‐ Vancouver Vancouver British Columbia Canada; ^4^ Department of Computer Science, Mathematics, Physics, and Statistics University of British Columbia‐Okanagan Kelowna British Columbia Canada; ^5^ Centre for Advanced Materials and Related Technologies (CAMTEC) University of Victoria Victoria British Columbia Canada; ^6^ Division of Radiation Oncology University of British Columbia Vancouver British Columbia Canada

**Keywords:** high dose rate brachytherapy, in vivo irradiations, pilot study

## Abstract

**Background:**

Pre‐clinical in vivo characterization is a necessary step in the translation of novel radiotherapeutic interventions to clinical application. In vivo irradiations using a radioactive source like Ir‐192 are challenging due to steep dose gradients and absence of universally available applicators that do not require physical contact or interstitial insertion.

**Purpose:**

To develop a novel external beam radiotherapy (EBRT) jig for accurate and reproducible radiotherapy treatments delivered using an Ir‐192 source for in vivo studies.

**Methods:**

An irradiation jig was constructed as a flat treatment bed and upright 6 cm diameter semi‐circle with eight peripheral catheter positions encompassing the lateral side of a mouse. A CT scan was acquired of the jig along with a silicone phantom of a mouse with flank tumor. The scan was imported into Oncentra® for planning using 500 cGy prescribed to the tumor and calculated using TG43 and collapsed cone. EBT4 film and OSLD measurements verified dose distributions in the axial and coronal directions along with the entrance and exit dose to the tumor. Monte Carlo simulations using TOPAS™ were used to observe the 3D dose distribution within the tumor itself. Five female immunodeficient mice inoculated with HEC‐1A cervical cancer tumors were irradiated and monitored for 3 weeks for tumor growth, body weight, and signs of toxicity.

**Results:**

Dosimetry measurements agreed within 2%–15% of the tumor's entrance and exit dose as reported by Oncentra® using both computational formalisms. Monte Carlo simulations confirmed a uniform dose distribution within the tumor of ± 10%. Compared to unirradiated mice, a significant reduction in tumor growth post‐irradiation was observed in all irradiated mice with no observable signs of toxicity.

**Conclusion:**

We have successfully developed an EBRT platform for in vivo irradiations with an Ir‐192 source. The platform can be adapted for various tumor and sample sizes and other radioactive sources.

## 1 INTRODUCTION

Pre‐clinical radiation studies are essential for establishing the safety and efficacy profiles of novel radiotherapeutic interventions prior to their translation into clinical workflows. In vitro pre‐clinical research using two‐dimensional and three‐dimensional cell cultures remains the current standard approach due to controlled experimental design,^1^, ^2^ However, results from in vitro systems often fail to translate into in vivo studies due to the complexity of biological systems that introduce additional variables,^2^, ^3^ In vivo radiation studies increase in complexity, as they must account for additional challenges such as radiation‐induced toxicity, tumor localization, animal immobilization, and pre‐ and post‐procedural care.

Beyond general considerations of in vivo radiation experiments, the choice of radiation delivery method introduces additional challenges. High‐energy external beam radiotherapy (EBRT) and brachytherapy (BT) in vivo delivery differ substantially in their technical demands when translated to animal models. For example, EBRT can be advantageous for in vivo irradiations due to more simplistic geometrical setups and collimation of the radiation field to the target volume. In contrast, BT delivery is more challenging due to an isotropic radiation source, and high dose fall‐off leading to highly heterogenous dose distributions within the tumor. Additionally, there are no known standardized methods for animal irradiations that avoid contact or interstitial placement of catheters in order to minimize trauma or infection risk to often immunocompromised models.^4^, ^5^, ^6^

This paper focuses on the development and characterization of a novel EBRT irradiation jig capable of accurate and reproducible dose delivery in vivo using a high dose rate (HDR) BT afterloader that poses minimal risk to the animal. The purpose of this research was to develop a simple in vivo irradiation platform capable of achieving homogeneous dose distributions to within ± 10% to tumor‐bearing NRG mice, enabling systematic investigation of novel therapeutic interventions in conjunction with a clinical HDR‐BT afterloader. A 3D‐printable jig was designed in two parts to connect to a clinical HDR‐BT afterloader and encompass the lateral side of a tumor‐bearing mouse without contact. An upright semi‐circle with eight guide channels for catheter placement and a flat treatment bed for easy tumor localization enabled EBRT irradiations with a clinical Ir‐192 source. The dimensions of the jig were carefully chosen through close collaboration with animal care specialists to ensure minimal contact between the animals and the jig, and minimal time under anesthesia. The jig was designed to be easily adaptable from the available CAD file, with generalizability to larger or smaller animal models with varying tumor sizes. Dose delivery to the defined target volume was validated using standard radiotherapy dosimetry tools: radiochromic film dosimetry, OSLD irradiations, and Monte Carlo (MC) simulations. Lastly, a preliminary pilot irradiation study with tumor‐bearing female NRG mice was conducted to demonstrate the jig's efficacy and short‐term safety profile.

## 2 METHODOLOGY

### 2.1 Jig design and development

The jig was first designed in CAD (SolidWorks) and then fabricated using polylactic acid (PLA) (*w_R_;* Hydrogen: 0.05595; Carbon: 0.50001; Oxygen: 0.44404)^7^ via 3D printing, which allowed for rapid prototyping during development and design iteration. During the design process, the following considerations were addressed to ensure the jig met both animal safety and dose delivery requirements.

1. **Length and Proximity of Source to Animal**: The length of the jig was chosen to completely encompass a lateral side of the mouse while accommodating variations in both mouse and tumor sizes. The proximity of the jig to the mouse was chosen to balance achieving a limited radiation dose fall‐off while also maintaining practical treatment times without requiring excessive anesthetic.
2. **Simple tumor Localization**: This ensured accurate dose delivery to the tumor and repeatability across experiments, leading to confidence in results.
3. **Minimal Contact with Animals**: This ensured animal exposure to unsanitized surfaces and areas was kept to a minimum during their time outside their cages.
4. **Non‐treatment site specific**: To ensure the radiation jig was accessible to as many centers as possible, the jig does not require additional components that are prostate, gynecological, breast, etc. specific in the event that one center does not have a working treatment program for that disease.

**FIGURE 1:**
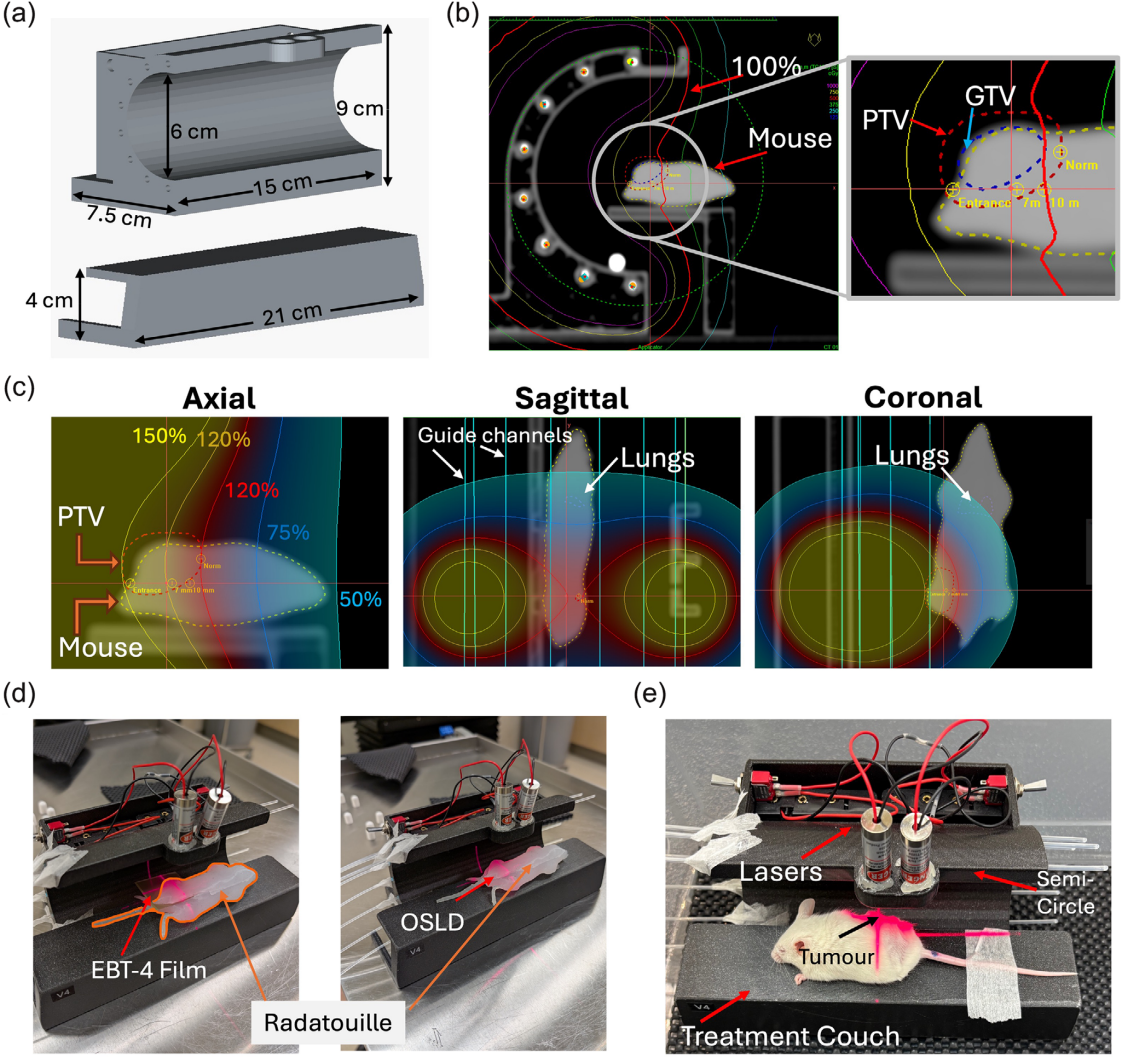
Design and validation of irradiation jig. (a) CAD file mock‐up with simple dimensions. (b) Treatment plan in Oncentra® treatment planning system with expanded view of target volume with the normalization point and three dose prescription points. (c) Axial, sagittal, and coronal plane of dose distribution between the 150%–50% isodose lines in Oncentra®. (d) 3D‐printed jig setup for irradiation and dose measurements with EBT‐4 film (left) and OSLD (right) placed within tumor volume of Radatouille (orange contour). (e) Mouse treatment delivery setup with tumor localized to the cross‐hairs of the axial and sagittal lasers.

The final design featured a semi‐circular structure 15 cm in length, 9 cm in height, and with a 6 cm diameter bore (Figure 1a). The structure was contoured to fit the lateral side of a mouse and included eight, equally spaced channels for catheters compatible with an HDR‐BT afterloader. A translatable couch was 3D printed using PLA to fit within the bore of the jig and aid tumor localization to the defined target region. The target volume and its normalization point was set to the center of the semi‐circle and mid‐point of the bore. Axial and sagittal lasers were mounted on the jig and aligned to intersect at this location to aid tumor localization. The couch was 4 cm in height and 21 cm in length which allowed it to translate into the bore of the jig. The CAD files of the semi‐circle structure and the treatment bed are available in the Supporting Information.

### 2.2 Treatment plannings

A CT scan of a tumor‐bearing mouse was acquired using a Siemens Inveon Multi‐Modality micro PET‐CT^8^ (Siemens Medical Solutions USA, Inc.) and was imported into the Eclipse Treatment Planning System™ (Varian Medical Systems, Palo Alto, USA). A body contour was created and exported from Eclipse™ as an STL file and cast with silicone inside a 3D‐printed PLA mould, using a modified version of BC Cancer Vancouver's 3D‐printed silicone bolus procedure.^9^ Several silicone models of the mouse were poured, herein referred to as Radatouille.

The 3D printed jig was CT‐scanned with a Radatouille positioned on its couch, and without the laser hardware mounted. A helical scan was acquired on a GE Discovery RT scanner (GE Healthcare Technologies Inc, Chicago, IL) using 0.06 cm slice thickness, 40 cm field‐of‐fiew, 440 mA and 80 kVp. The scan was registered to the CT scan of the tumor‐bearing mouse and the tumor, heart and lungs were contoured on Radatouille in order to estimate organ‐at‐risk (OAR) dose. The contoured scan was imported into Oncentra® (Figure 1b) for planning. A 5 mm PTV margin was added to the tumor (GTV) to account for setup variation and tumor size variability between animals. A normalization point was added to the medial most extent of the tumor along the axial slice in line with the center of the jig. Three dose assessment points for subsequent dosimetric comparison were added to determine the 1: Entrance Dose, 2: Dose at 7 mm depth, and 3: Dose at 10 mm depth medially (Figure 1b). Full isodose lines in the axial, sagittal, and coronal planes in line with the dose prescription points and normalization point are presented in Figure 1c. The generated treatment plan was used for all subsequent in vivo irradiations. Each mouse did not receive an individualized treatment plan.

Along each of the eight channels, seven equally spaced dwell positions, 2 mm apart, were centerd along the jig. Each position received the same weight and dwell time to achieve the prescription dose to the normalization point. PTV dose was recorded according to a commissioned model‐based dose calculation algorithm, ACE® (Advanced Collapsed‐cone Engine), and TG‐43 formalism. OAR doses were recorded by TG‐43 and not ACE® to facilitate more efficient calculation times. ACE® was commissioned for research applications per the TG‐186 level one guidelines.

### 2.3 Plan delivery validation

Treatment plan delivery was accomplished using an Ir‐192 Flexisource and an Elekta Flexitron HDR Afterloader (Elekta, VEENENDAAL, Netherlands). The eight specified channels from the afterloader were attached to Eight catheters with collars (designed for use with Elekta's Multi Channel Vaginal Applicator) were inserted into the eight jig channels and connected to the afterloader via transfer guide tubes. The jig was designed such that the catheter collars stopped once flush to the entrance of each channel and the catheters were taped in place to ensure they could not move during source translation.

An incision was created in the coronal plane of the tumor on Radatouille's left flank at the height of the dose assessment points previously described in Section 2.2. EBT‐4 radiochromic film (Lot #: 11222401) was cut into a 1.5 × 1.5 cm square sheet and fit within the incision (Figure 1d). Radatouille was placed on the treatment bed and its tumor localized to the center of the jig using both the axial and sagittal lasers. Post‐irradiation, the film was left to equilibrate for 24 hours. We used FilmQAPro software (Ashland, Advanced Materials, Bridgewater, NJ) to analyze the film dose using the Red Channel for dosimetry. Film calibration was based on a Clinical Standard Protocol recommended by the AAPM TG‐235 Radiochromic Film Dosimetry with a 6 MV photon beam from a linear accelerator (LINAC) (Varian TrueBeam) with calibration doses ranging from 0 to 8 Gy.^10^ Similarly, an OSLD (MyOSLchip, Radpro International GmbH, Remscheid, Germany) was placed within the incision and irradiated to the same prescription dose and left for 60 min prior to read‐out.^11^ We used a microSTARi reader (Landauer, Inc., Glenwood, IL) to measure the dose received by the OSLD. Calibration was also performed using a Clinical Standard Protocol as recommended by the AAPM TG‐191,^12^ calibrating it with a 6 MV LINAC photon beam with calibration doses ranging from 0 to 10 Gy. Film readings and OSLD measurements were then compared to the predicted dose by ACE® and TG‐43 at the three prescription points.

### 2.4 Monte Carlo simulations

MC simulations were performed to verify tumor dose delivery and dose distribution within the tumor itself. All simulation were conducted with Tool for Particle Simulation (TOPAS) v3.9.^13^ Berumen et al. previously validated TOPAS for HDR‐BT applications following TG‐186 recommendations and compared to reference data (MCNP6 MC code for source validation, and ALGEBRA MC code for clinical cases).^14^, ^15^, ^16^ They reported air‐kerma strength within 0.3% of reference, dose‐rate constant within 0.01% of reference, and local dose differences within ± 1% for at least 96.9% of voxels across all TG‐186 test cases. Global dose differences were within ± 1% for at least 97.5% of voxels across clinical geometries of the prostate, breast, and lung.

An STL file of the jig along with Radatouille was exported from Oncentra® and imported into TOPAS for accurate treatment setup, with their material both set to water (G4_Water). A box of air (G4_Air) was set to encompass the tumor of the mouse for dose scoring. During geometry overlap between Radatouille and the box, the material of the mouse was set to take priority for the purpose of dose scoring. The box was set as a 2 × 2 × 3 cm voxelized object with 0.5 mm/voxel, and the total dose to each voxel was scored. The physics module used was the “g4em‐standard‐opt4” package. Default cutoff for all particles in the simulation was set to 50 µm, and photon‐electrons interactions are modeled down to 100 eV.

A Flexisource Ir‐192 source was modeled as a generic MBDCA‐WG source (Ir‐192 model file provided by Berumen et al.^16^).^17^ The radiation source was modeled as a flat, ellipsoid beam emanating from the active source volume (G4_Ir), with an initial photon spectrum from the National Nuclear Data Center matching that of Ir‐192.^18^ The beam had an angular cutoff between ± 90° in the X and Y dimensions. The angular rotation of the beam was changed for each guide channel to be directed towards the target volume to facilitate more efficient simulations as opposed to an isotropic radiative source.

All source dwell positions were simulated according to their position as reported by Oncentra®. The total simulation was split into 56 individual simulations corresponding to unique dwell positions. For each simulation the source was translated in between the defined dwell positions. The number of histories was set to 5 × 10^7^ for each dwell position. Each simulation was run on an Intel® Xeon® Silver 4210R CPU with 10 cores each and 2 threads (20 threads total). The simulations took approximately 30 hours to complete in total. The dose per voxel was summed over all simulations and the total dose was processed in Python v3.11.2 on a personal computer. The dose to each voxel was then normalized to the geometrical position of the normalization point, which was set in Oncentra® (see Section 2.2, Figure 1b). The intersection of the box with the STL file of the GTV was then kept as the representative dose to the tumor volume, while all other dose was removed from the dataset. The mean dose, 2‐D dose distribution in the axial, coronal, and sagittal planes, and their dose profiles were then recorded and compared to Oncentra® reported doses, and EBT‐4 and OSLD measurements. MC uncertainty in the dose profiles was calculated as a combination of the statistical uncertainty of 8 mm regions of interest centered on the profile and MC uncertainty calculated in TOPAS.^16^, ^17^

### 2.5 Pilot irradiation study

Ten female NRG mice were purchased from the BC Cancer Research Institute Animal Resource center (ARC, Vancouver, BC, Canada). The right flank of mice was injected with 5 × 10^6^ Human Endometrial Carcinoma – 1A (HEC‐1A) cells subcutaneously in a volume of 50 µL using a 28‐gauge needle. The mice were inoculated at a time such that once the tumors reached approximately 250–300 mm^3^, the Ir‐192 source activity was sufficiently high to allow for fast irradiations.

The mice in the irradiation group were then transported to the cancer center in covered cages after patient‐treatment hours to limit viewing of the animals. In addition, the entrance to the HDR‐BT treatment unit was covered with a barricade to prevent patients or other staff from observing the animals. The jig was placed on top of a high friction mat to prevent slipping, which was all placed within a plastic container as a precaution to prevent the mice from escaping. Eight holes were drilled into the box to allow for catheter placement into the channels of the jig.

Mice were anaesthetized one at a time approximately 3–5 min before planned irradiation and were then individually brought into the treatment vault where the irradiation jig had been setup. The mouse was placed onto the treatment bed and its tail was taped down to prevent movement should it prematurely recover from the anesthetic (Figure 1e). Once immobilized, the bed was translated to localize the tumor to the center of the jig using the onboard lasers. In the case of oblong or irregularly shaped tumors, the estimated center of mass of the tumor was used as the localization point, while also ensuing the tumor remains within the estimated PTV location. Post‐irradiation, the mice were brought back to the cages where the animal care specialist carefully monitored their behavior until the anesthetic wore off. After all irradiations were complete, the mice were transported back to the animal care facility for monitoring of tumor volume, bodyweight, and signs of toxicity. Mice bodyweight change is reported relative to the tumor inoculation date. Mice were euthanized once the tumors reached approximately 800 mm^3^.

### 2.6 Statistics

OSLD measurements were conducted in duplicate. Tumor growth trends and mice bodyweight differences were compared between groups using a linear mixed effects model with an interaction term. The group, day, and interaction term (group *x* day) were fixed effects, while mouse was included as a random effect with both random intercept and random slope. The model compared both the effects of group and day, as well as whether the rate of tumor growth or body weight change differed between groups. Endpoint‐free survival of the two mice groups was evaluated using a log‐rank test, where the defined endpoint event was planned euthanasia upon tumor volume reaching 800 mm^3^. Significance was defined as *𝛂 =* 0.05.

## 3 RESULTS

### 3.1 Plan delivery verification

**FIGURE 2:**
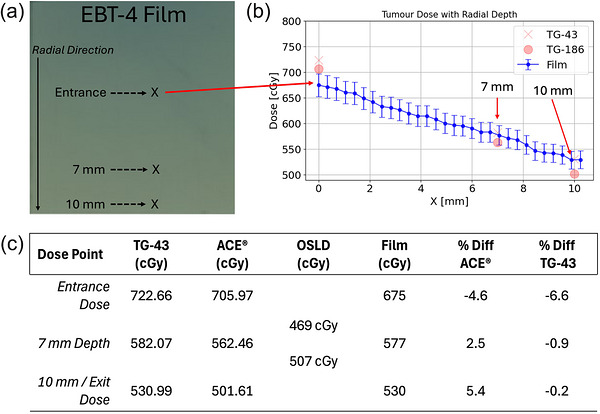
(a) Irradiated EBT‐4 film with the entrance dose, 7 mm depth, and 10 mm depth locations marked. The radial direction is also shown relative to the Ir‐192 dwell positions. (b) Central axis tumor dose as a function of depth with labeled dose reference points from TG‐43 and ACE®. (c) Absolute dosimetry of film and OSLD compared to reported doses by TG‐43 and ACE® for the Entrance Dose, 7 mm Depth, and 10 mm Depth.

A 500 cGy treatment prescription to the normalization point resulted in an average dose to the tumor of 596 cGy (ACE®) and 615 cGy (TG‐43). The heart and lungs received an average of 260 cGy and 259 cGy, respectively, according to TG‐43. Measured EBT‐4 dose profiles in the radial direction along with OSLD readings were consistent with the predicted dose values from TG‐43 and ACE® (Figure 2). The measured film response shows consistent dose fall‐off over the tumor region as expected (Figure 2a,b). Error bars are given by the approximate dosimetric error in the red channel when using a 6 MV calibration curve for Ir‐192 irradiations.^19^ Entrance dose to the tumor was 35% higher compared to the 500 cGy prescription dose due to the normalization of the plan at the medial most point of the tumor, furthest from the radioactive source. Compared to ACE® and TG‐43, measured film response was approximately −5% to 5% predicted dose values (Figure 2c). Due to the imprecise nature of OSLD localization within the cut slit in Radatouille, its measured dose value was compared to the 7 mm depth dose prescription point. Compared to ACE® and TG‐43, average OSLD measured dose reported within −13.2% and 16.2%, respectively.

### 3.2 Monte Carlo simulations

**FIGURE 3:**
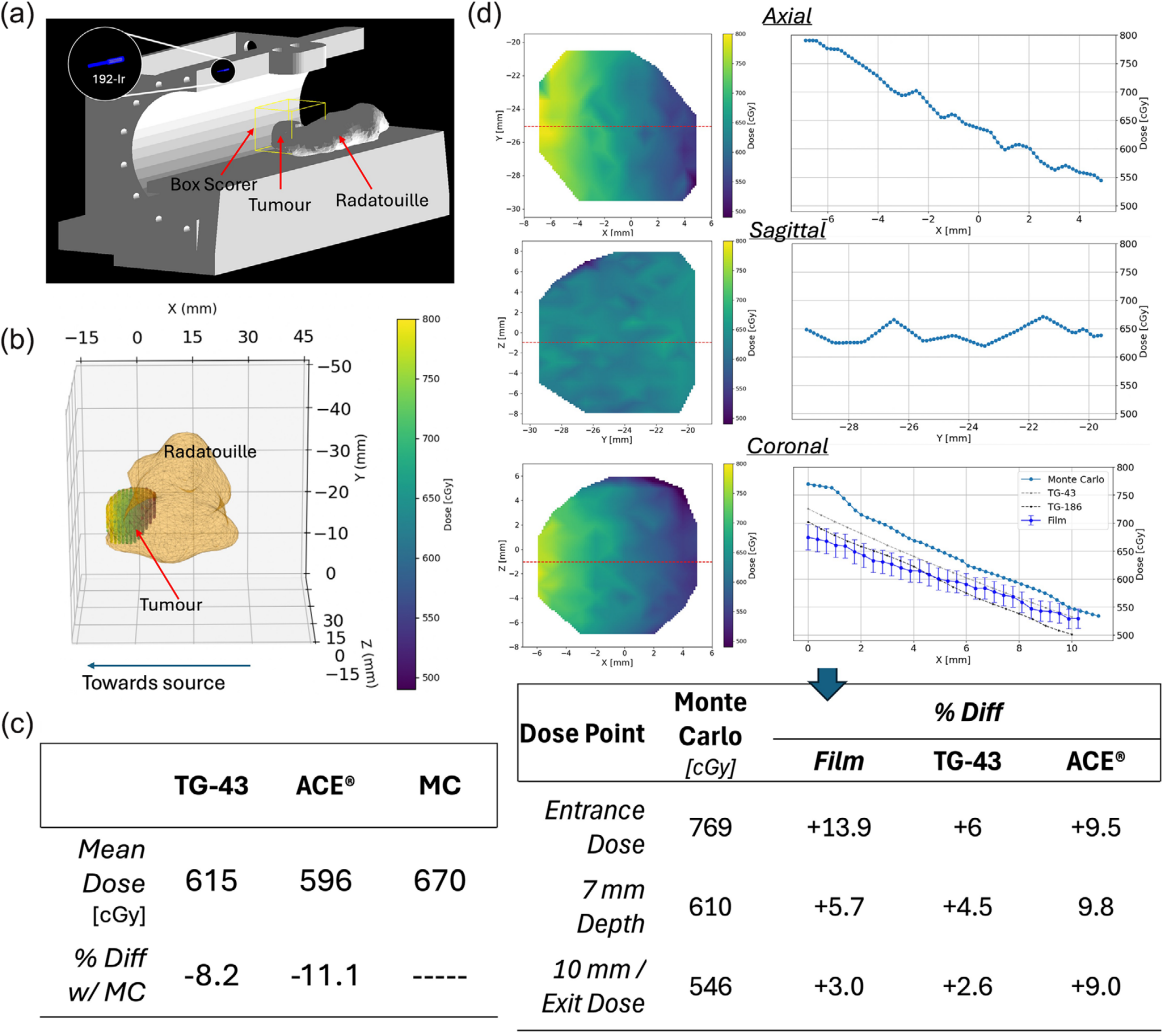
(a) Monte Carlo (MC) geometrical setup with the scorer as the box‐of‐air surrounding the tumor volume. (b) 3D dose distribution within the tumor volume. (c) Mean reported dose by TG‐43, ACE®, and MC. (d) 2D and 1D dose distribution in the axial, sagittal, and coronal direction at the height and depth of the reference dose points defined in Oncentra®.

Monte Carlo simulation setup is shown in Figure 3a, and representative 3D dose distribution within mouse tumor volume in Figure 3b. The mean dose to the tumor volume was reported as 670 cGy ± 70.4 cGy, 12.4% and 8.9% greater than the mean reported dose by ACE® and TG‐43, respectively (Figure 3c). Dose fall off in the axial, sagittal and coronal plans at the height of the dose prescription points (see Section 2.3) is shown in Figure 3d. In the axial plane, the dose fall‐off resulted in a decrease in ∼250 cGy from entrance to exit dose. In the sagittal plane, no dose fall‐off is measured with the median dose across the slice approximately 632 cGy ± 9.8 cGy. Lastly, in the coronal plane at the height used for film and OSLD dosimetry, dose fall‐off resulted in an approximate decrease of 223 cGy (22.3 cGy/mm), compared to only 130 cGy (13 cGy/mm) as measured using EBT‐4 film (EBT‐4, ACE®, and TG‐43 dose fall off also shown in the coronal profile in Figure 3d for comparison). From TG‐43 and ACE®, dose fall‐off agreed well with MC, reporting an average dose fall‐off of 20.2 cGy/mm and 20.1 cGy/mm, respectively.

The triangular patterns observed in the dose distribution within the tumor volume are likely a consequence of how TOPAS processes STL geometry. An STL file encodes only triangular surfaces without volumetric information. When TOPAS imports the STL object, Geant4 reconstructs the interior by voxelizing the closed triangular mesh. This voxelization can result in planar regions or boundaries aligned with the STL triangles, which could lead to local alterations of scattering behavior and local material density. These artifacts only occupy small regions of space within tumor and do not affect the global dose distribution or metrics used in this study.

### 3.3 In vivo irradiations

The source activity at the time of irradiation was 9.8 Ci, immediately post source exchange. For a 500 cGy treatment prescription, each dwell time were set to 12.3 s which resulted in a total irradiation time of 689 s per animal, not including time for dummy cable checks and source transit. Four of the five irradiated mice tolerated the treatment well, with no observable signs of stress during or immediately post radiation delivery. One mouse appeared to suffer from a heart attack early on during radiation delivery; however, this was not expected to be from the radiation delivery but rather a response from the anesthetic as indicated by the on‐site animal care specialists. Post‐treatment the mouse recovered and was transported back to the animal care research center and was included in the post‐delivery analysis.

**FIGURE 4:**
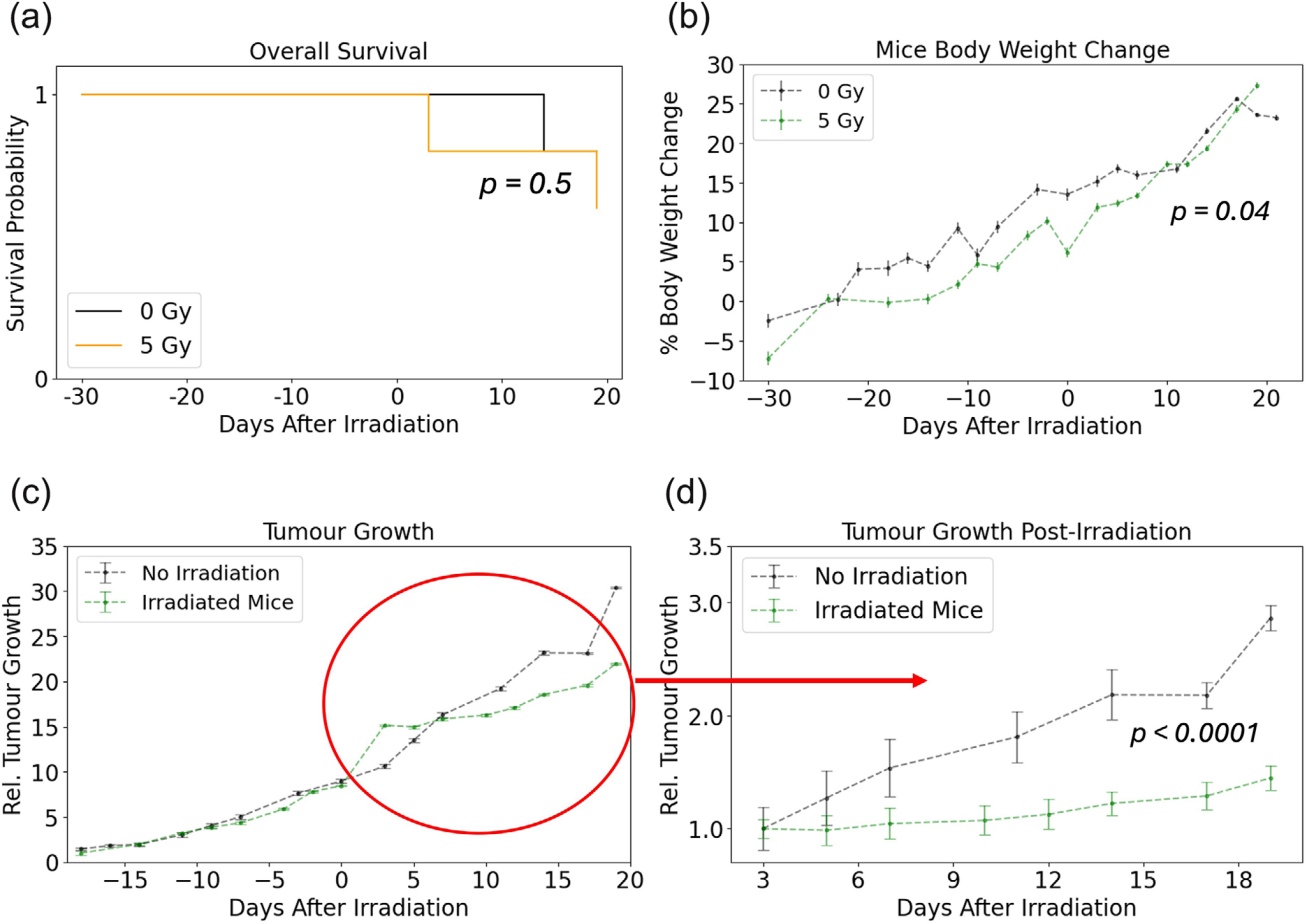
(a) Overall survival of mice pre‐and post‐irradiation. (b) Mice body weight change with and without radiation. (c) Relative tumor growth as a function of time without (black) and with (green) irradiation. (d) Relative tumor growth of both unirradiated (black) and irradiated (green) mice groups as a function of time 3 days post‐irradiation.

Post‐irradiation, the mice were monitored for a total of nineteen days. One irradiated mouse was euthanized pre‐maturely at day three post‐irradiation due to excessive tumor growth. This mouse was not included in the irradiated tumor volume group due to the anomalous behavior. No mice exhibited signs of radiation induced toxicity (Figure 4a). The rate at which mice bodyweight increased in the irradiated group was slightly greater (*p* = 0.04) than in the unirradiated group (Figure 4b); however, it is unknown whether this is an abnormal healthy tissue response or an anomalous measurement due to the low sample size of N = 5 per group.

Tumor volume changes pre‐radiation delivery between the two groups was not significantly different (Figure 4c). Post‐radiation delivery, a sharp increase in tumor volume is measured in the irradiated group, likely due to radiation‐related edema; however, this was not verified. Between three to sixteen days post‐irradiation, tumor growth is stunted in the irradiated group compared to the unirradiated group (*p < 0.0001*) (Figure 4d). Relative tumor growth in the irradiated group was reduced considerably by 280% after 19 days compared to 19 days pre‐irradiation, and by 49.3% relative to tumor growth in unirradiated mice.

## 4 DISCUSSION

In vivo irradiations can help translate novel therapeutic interventions to the clinic, potentially improving curative rates or reducing normal tissue toxicity. However, pre‐clinical in vivo irradiations with a radioactive source like Ir‐192 are difficult due to complex geometric setup and confidence in delivery accuracy. Therefore, pre‐clinical research involving in vivo irradiations using Ir‐192 is rarely conducted. For example, gold nanoparticles (GNPs) as radiosensitizing agents for HDR‐BT show promise in vitro, but require in vivo experimentation before clinical translation.^20^, ^21^ The purpose of this research was to develop an adaptable, user‐friendly method for external beam in vivo irradiations using a clinical Ir‐192 source and HDR‐BT afterloader, which is distinct from invasive intratumoral procedures or brachytherapy program‐specific applicators like a bronchial sleeve.^4^, ^6^ The jig was designed to be compact, with simple tumor localization to the treatment volume without excessive skin‐to‐jig contact, and to be site‐independent, requiring no pre‐existing HDR‐BT infrastructure other than a treatment vault and access to an HDR‐BT afterloader. To the authors’ knowledge, no comparable platform has previously been described.

The treatment delivery was verified to be successful and accurate, measured using radiochromic film and OSLDs. Radiochromic film measurements were calibrated using a 6 MV calibration curve, as the delivered dose exceeded the recommended threshold for Ir‐192 irradiations.^19^ Uncertainty in the dose measured via radiochromic film is estimated to be approximately 3.3%, though this is not expected to significantly affect our presented results.^19^ The OSLDs were previously shown to exhibit a dosimetric accuracy of approximately 5%.^11^ These uncertainties remain within an acceptable range when considering pre‐clinical irradiation studies with a high activity radioactive source. It is recommended that OSLD or similar dosimetric measurements within the target volume be conducted for the adaptation of the jig to different animal or tumor sizes. This would require the fabrication of a silicone animal model similar to the Radatouille. Dose fall‐off measured via EBT‐4 film exposure was less than predicted by TG‐43 and ACE® at approximately 13 cGy/mm compared to 20.2 and 20.1 cGy/mm, respectively. This discrepancy may be due to two factors. First, the placement of the film within the incision of Radatouille may not have been exactly horizontal or in the exact plane of the dose prescription points, which could sample a shallower dose gradient. Second, the nature of the incision reduces the scattering medium around the film, potentially reducing the gradient relative to predictions by Oncentra®.

Treatment planning was conducted using both TG‐43 and ACE® formalisms to enable a comparison between water‐based dose calculations and a collapsed cone algorithm incorporating tissue heterogeneity. Interestingly, when ACE® was used to calculate the delivered dose, the mean dose dropped compared to TG‐43, despite lower attenuation through the air‐gap from the jig to the mouse which would be presumed to be water with TG‐43. It is hypothesized that this is due to reduced scattering from the air relative to water and not based on incorrect treatment setup or delivery errors. Furthermore, all material in TOPAS, other than the source and its encapsulation, was set to water, which could also lead to a closer match with TG‐43 compared to ACE®, which considers material density differences. It is recommended that for the adaptation of this jig to larger or smaller animal models, treatment delivery is planned using model‐based algorithms due to the large air‐gap between the source and target volume. However, our results indicate TG‐43 formalism is sufficient to ensure accurate dose delivery from the jig with the given air‐gap.

MC simulations agreed well with predicted delivered dose from Oncentra® as well as measured dose from EBT‐4 film and OSLD. MC predicted dose fall in the coronal plane was larger compared to the measured dose fall off with EBT‐4 film, and that predicted by TG‐43 and ACE®. The placement of the film within an incision into the tumor of the Radatouille, which could have reduced the effective attenuation through the film, reducing the measured dose gradient. Furthermore, the film may have recorded lower absolute dose values compared to that predicted by MC due to reduced scatter contribution under these conditions. Additionally, the irradiation jig and platform were simulated as water, while the true material was fabricated from PLA, which may result in greater scatter and attenuation during experiments compared to simulations.

The offset between MC and TG‐43/ACE® mean tumor dose and fall‐off is hypothesized to arise due to voxel averaging effects. Dose in TOPAS was scored on a 0.5 mm/voxel grid, while Oncentra® calculates dose at geometric points. Therefore, normalizing the MC dose to the voxel containing the normalization point could introduce scaling errors proportional to the dose gradient in the voxel. Minor modifications to the mouse body STL file was required prior to TOPAS import to correct for gaps and sharp edges that cause stuck particle tracks. These modifications may have contributed slight differences in scatter conditions.

The pilot irradiation study was included in the testing of the novel radiation jig to demonstrate its efficacy and safety profile in vivo using a therapeutic dose prescription. In vivo delivery was successfully achieved with minimal observed side effects. No radiation induced toxicity was observed in the mice despite no active shielding applied up to the experiment endpoint; this decision was made to limit contact between the mouse and unsanitary working surfaces, though did not appear to affect their overall condition post‐radiation delivery. Significant tumor growth delay was observed after treatment delivery, as expected, indicating that the tumor was successfully targeted (Figure 4d). The 500 cGy treatment prescription was given to align with current HDR‐BT practices, while maintaining dose limits to the mice.^22^ However, given the limited tumor growth measured in the irradiation group, a lower treatment prescription could be delivered for future efficacy studies to ensure proper therapeutic effect of novel interventions is measured. For the expansion of this platform into additional tumor types and treatment locations, tumor growth should first be evaluated in a pilot study to determine the optimal dose prescription. Furthermore, when inoculations sites are located near critical organs at risk (e.g. heart, lungs, brain), the delivered dose to these structures should be carefully evaluated, which was not done in this study. Although no toxicity was observed in the weeks immediately following radiation delivery, longer‐term studies are warranted to assess potential delayed radiation effects. In the present study, the experimental endpoint was defined as a tumor volume exceeding 800 mm^3^; however, variability in tumor growth rates across models could prolong study duration and increase the likelihood of observing radiation‐associated toxicity.

One of the largest considerations made for treatment planning and delivery was the activity of the radioactive source at time of delivery. When coordinating with the animal care specialists regarding treatment delivery time, it was noted that a low (∼4.5 Ci) to medium (∼7 Ci) source strength could result in excessive treatment delivery times and would require more anesthetic that could affect the health of the mice. Note that this was not directly tested, and a cold source may still be viable for future studies if the health of the mice is not compromised by the level of anesthetic injected. Listed in Table 1 below are treatment delivery times for various dose prescriptions and source activities acquired from Oncentra®; additional dummy source cable checks adds 6.5 min to the listed times. In consultation with the animal care specialists, the amount of anesthetic delivered to the mice should not exceed ∼25 min = Treatment Delivery [min] + Dummy Cable Check [min]. Therefore, when adapting the jig to larger tumor sizes or animal models, careful consideration should also be made to the treatment delivery time and anesthesia requirements of the animal with larger treatment prescriptions and larger source‐to‐sample distances.

**TABLE 1:** Treatment delivery times as function of both dose prescription and source activity at a fixed source‐to‐sample distance.

Dose [cGy]				
Activity (Ci)	300	350	400	500
	Treatment delivery [min]			
10	14	15	16	18
9	15	16	17	20
8	15	17	18	21
7	17	18	20	23
6	18	20	22	26
5	21	23	25	30

Lastly, to expand the jig to larger or smaller animal models, we recommend a full CT scan of the newly printed radiation jig for accurate treatment planning purposes along with accurate dosimetric analysis similar to that conducted with Radatouille. As noted previously, adapting the jig to larger animals can greatly increase the treatment delivery time, which will need to be discussed with local animal care specialists to ensure the health and safety of the animals. Expansion to other tumor inoculation sites than the flank will need additional shielding considerations due to possible radiation exposure to critical OARs like the heart and lungs.

## 5 CONCLUSION

In vivo irradiations improve the clinical translatability of novel therapeutics by measuring their efficacy in a biological environment. To the authors knowledge, this research is the first of its kind for a novel EBRT jig capable of accurate in vivo irradiations from an Ir‐192 source using an HDR‐BT afterloader. The jig is modular and could be expanded to larger or smaller animal sizes through simple adjustment to the CAD file. The delivery accuracy of the jig was verified and confirmed using radiochromic film and OSLD measurements. Efficacy of the jig was evaluated using tumor‐bearing female NRG mice and showed significant tumor growth delay in irradiated compared to unirradiated groups, with no observable signs of toxicity from radiation exposure to healthy tissue. This work represents a first step toward enabling pre‐clinical evaluation of the safety and efficacy of emerging therapeutics for use with Ir‐192 irradiations.

## CONFLICT OF INTEREST STATEMENT

The authors declare no conflicts of interest.

## Supporting information




**Supporting Information**: mp70540‐sup‐0001‐SuppMat.pdf

## Data Availability

The datasets used and/or analyzed during the current study are available from the corresponding author on reasonable requests.
