# Supplementary material for: Development and characterization of a novel, small animal external beam irradiator using a clinical high dose rate brachytherapy source
Source: Med Phys. 2026 Jun 30;53(7):e70540. doi: 10.1002/mp.70540 (PMC13316811; doi:10.1002/mp.70540)
Supplement: Supplementary file 1 — Supporting Information: mp70540‐sup‐0001‐SuppMat.pdf [file MP-53-0-s001.pdf]

Development and characterization of a novel, small animal external beam irradiator using a clinical high dose rate brachytherapy source

Daniel Cecchi[1,2], Sacha Freeman[1,2], Greg Warren[3,4], Brad Gill[3], Chris Johnstone[3], Devika B. Chithrani[1,2,5], Samantha AM Lloyd[3,6]*

1. Department of Physics and Astronomy, University of Victoria, Victoria, BC, V8P 5C2, Canada.
2. BC Cancer - Victoria, Victoria, BC, V8R 6V5, Canada.
3. BC Cancer - Vancouver, Vancouver, BC, V5Z 4E6, Canada
4. Department of Computer Science, Mathematics, Physics, and Statistics, University of British Columbia-Okanagan, Kelowna, BC, V1V 1V7, Canada
5. Centre for Advanced Materials and Related Technologies (CAMTEC), University of Victoria, Victoria, BC, V8P 5C2, Canada.
6. Division of Radiation Oncology, University of British Columbia, Vancouver, BC, V5Z 1M9, Canada

*Corresponding Author: devikac@uvic.ca

GITHUB Repository for In Vivo External Beam Radiotherapy Jig

https://github.com/danieldcecchi/In-Vivo-External-Beam-Radiotherapy-Jig
